# Small populations, big impact: leveraging rare disease gene therapies to benefit millions

**DOI:** 10.1172/JCI212036

**Published:** 2026-10-01

**Authors:** Stephanie Cherqui

**Affiliations:** Department of Pediatrics, Division of Genetics, University of California, San Diego, La Jolla, California, USA.

Rare diseases, although individually uncommon, collectively affect millions of individuals worldwide and represent a major unmet medical challenge. More than 7,000 rare disorders have been identified, together impacting over 300 million people globally, highlighting that their cumulative impact represents a major global health burden. The vast majority are caused by genetic mutations, where disruption of a single gene can result in devastating consequences, including metabolic dysfunction, blindness, myopathy, neurodegeneration, multiorgan failure, and premature death. Despite their severity, therapeutic options for most rare diseases have been limited or entirely absent.

The concept of gene therapy emerged more than five decades ago through the visionary work of Theodore Friedmann and colleagues on Lesch-Nyhan syndrome, who first proposed the possibility of treating disease by introducing functional genetic material into cells (1). What began as a theoretical framework has evolved into a rapidly advancing clinical field driven by major technological breakthroughs. Today, a broad range of genetic medicines, including adeno-associated viral (AAV) vector–mediated gene replacement, lentiviral gene transfer, antisense oligonucleotides, RNA interference, and CRISPR/Cas-based genome editing, are transforming the treatment landscape for rare diseases. Several of these approaches and their applications are summarized in Figure 1.

Importantly, rare diseases have be-come a proving ground for innovative therapeutic platforms, motivated by the severe nature of many of these conditions. Despite setbacks, unexpected adverse events, manufacturing and delivery challenges, and intense regulatory scrutiny, advances achieved in rare diseases are already delivering life-changing therapies for previously untreatable conditions. At the same time, these successes are establishing therapeutic principles and technological platforms that can ultimately be extended to more common disorders. As genetic medicines continue to mature, the lessons learned from rare disease research are likely to shape the future of precision medicine across a broad spectrum of human disorders.

## The risks, the press, and the progress

The path toward advanced genetic therapies for rare diseases has not been linear. Alongside remarkable successes, the field has faced considerable setbacks that underscore the risks of innovation. When complications arise, they often receive substantial media attention, fueling widespread concerns and, at times, slowing scientific and regulatory progress. While media coverage of adverse events is understandable given the novelty and potential risks of gene therapy, reactions to this coverage can at times be disproportionate, causing periods of hesitation that delay progress for patients with rare diseases who often have few or no therapeutic options. Each adverse event has also provided critical lessons that have ultimately strengthened the field.

A major early setback in gene therapy occurred in 1999, when 18-year-old Jesse Gelsinger died four days after receiving an experimental adenoviral vector for ornithine transcarbamylase deficiency (2). His death marked a turning point for the field, leading to increased regulatory oversight, stricter clinical trial requirements, and a renewed emphasis on vector safety and immune responses. Although devastating, this event forced the community to reassess assumptions and improve the design and monitoring of viral gene therapy trials.

Subsequent challenges emerged in early clinical trials for X-linked severe combined immunodeficiency (SCID-X1), where several patients developed leukemia due to insertional mutagenesis associated with **γ**-retroviral vectors (3, 4). These events temporarily halted progress in the field of gene therapy, despite demonstrating the potential for sustained immune reconstitution and clinical benefit (5). However, these events also drove the development of safer self-inactivating vectors and lentiviral vectors, which demonstrated improved safety and integration profiles (6). Today, lentiviral-based gene therapies have become foundational tools in treating multiple genetic disorders.

More recently, AAV-based therapies, while transformative for many conditions, have also been associated with toxicities in certain clinical contexts. Serious adverse events, including hepatotoxicity, thrombotic microangiopathy, dorsal root ganglia toxicity, myositis, cardiopulmonary failure, and other consequences, including death, highlight important limitations related to vector dose, capsid tropism, and host immune responses. For example, high-dose systemic AAV administration in trials for X-linked myotubular myopathy (XLMTM) was associated with fatal liver dysfunction in several patients (7), halting clinical trials. These events have accelerated efforts to engineer safer capsids, develop more efficient vectors requiring lower doses, and gain a better understanding of immune and organ-specific toxicities associated with systemic AAV delivery.

Therefore, challenges encountered in gene therapy trials have driven technological innovation, strengthened regulatory oversight, and improved our understanding of gene delivery biology, including host responses, paving the way for safer and more effective therapies.

## Successes for untreatable disorders and the path forward

Despite early setbacks, viral gene therapy has entered a period marked by clinical successes that have renewed hope for patients living with previously untreatable genetic disorders. A major milestone in the field was the approval of Luxturna by the US FDA in 2017. Luxturna was the first viral gene therapy product for a rare inherited disease, restoring vision in patients with Leber congenital amaurosis after subretinal injection of an AAV containing a functional copy of the retinal enzyme-encoding gene *RPE65* (8). Since then, additional AAV-based therapies have attained FDA approval, including Zolgensma for spinal muscular atrophy. Zolgensma has demonstrated remarkable benefits in terms of survival and motor function, including improving the ability of some treated children to sit, stand, and walk when therapy is administered early (9). More recently, the approval of Otarmeni, which restores hearing in children with otoferlin deficiency–related deafness, further demonstrated the expanding therapeutic potential of viral gene delivery approaches for sensory disorders (10).

Ex vivo hematopoietic stem cell (HSC) gene therapies have also achieved major clinical successes. Although the number of approved products remains limited, notable examples for inherited immune disorders include Strimvelis for adenosine deaminase–deficient severe combined immunodeficiency (ADA-SCID), which utilizes a **γ**-retroviral vector (11), and more recently Kresladi for pediatric patients with severe leukocyte adhesion deficiency type I (LAD-I) using a lentiviral vector (12). Ex vivo HSC gene therapy has also shown transformative benefits in neurodegenerative disorders such as metachromatic leukodystrophy, where Libmeldy provides durable survival and neurological improvements when administered at early stages of disease (13). In addition, the approval of Cavesgy a CRISPR/Cas9-mediated ex vivo HSC therapy for sickle cell disease and β thalassemia marked a milestone for genome editing as a therapeutic modality (14). Unlike gene addition strategies, genome editing enables precise modification of the endogenous genome, offering the potential for more physiologic gene regulation and an improved safety profile. Nevertheless, careful assessment of off-target editing and genomic integrity remains essential to ensure the safety of these therapies.

The histories of these successes underscore the magnitude of the challenges that remain. Each approved therapy represents years of intensive toxicology and pharmacology studies, highly specialized and costly manufacturing processes, and extensive regulatory documentation. The complexity and expense of this pathway render bench-to-bedside translation exceptionally difficult, particularly for rare diseases with small patient populations, and result in considerable cost for the products. As a result, many promising therapeutic concepts lack a conventional business rationale and are then delayed, abandoned, or never initiated despite strong scientific rationale. As an example, despite more than four decades of research and the demonstrated clinical efficacy of HSC gene therapy for ADA-SCID, Orchard Therapeutics discontinued its commercial development efforts in both Europe and the United States due to insufficient commercial viability, and the associated rights were subsequently returned to the originating academic institutions (15).

Accelerating the development and approval of gene therapy products will be critical to expanding their impact. Regulatory programs to expedite therapies for rare diseases, such as Orphan Drug, Fast Track, Breakthrough Therapy, and Regenerative Medicine Advanced Therapy designations by the FDA, have helped streamline development. Emerging regulatory innovations, including the Plausible Mechanism Framework, may further shorten timelines by enabling approval based on mechanistic understanding supported by early clinical evidence. Additional innovations are needed to reduce the time and cost required to bring gene therapies to patients. Platform-based manufacturing approaches, umbrella investigational new drug applications, and master clinical trial protocols could enable multiple diseases that share common technologies, manufacturing processes, and development strategies to be evaluated within a unified regulatory framework. Such approaches have the potential to substantially improve development efficiency, lower costs, and expand patient access to transformative genetic medicines. This shift will require acceptance of a risk-based development paradigm, in which prior knowledge from well-characterized drug products can reduce redundant testing while relying on more targeted product-specific safety evaluations.

Perhaps the most extraordinary demonstration of what is possible when science, regulation, and clinical urgency align is the recent case of an infant known as Baby KJ, who received a personalized base-editing therapy within nine months of diagnosis, a timeline that contrasts sharply with the six or more years typically required to advance a gene therapy to the clinic. This unprecedented achievement raises important questions: What infrastructure, regulatory flexibility, and collaborative frameworks enabled this progress? And more importantly, how can such an approach be scaled so that individualized, life-saving therapies become the norm rather than rare exceptions?

These success stories affirm that genetic therapies are no longer a distant promise but an emerging reality. The challenge ahead is not only to replicate these achievements, but to redesign development pathways so that transformative therapies can reach many more patients with rare diseases and to do so within timelines that match the urgency of their needs.

## Rare disease research catalyzes innovation in common diseases

Innovations driven by rare disease research have catalyzed technical advances to provide therapies for more common conditions. Technologies developed for rare genetic disorders, including lentiviral gene transfer, gene editing, and ex vivo hematopoietic stem cell manipulations, have directly enabled transformative treatments such as CAR T cell therapies. In this personalized cancer immunotherapy, a patient’s own immune cells are genetically engineered to recognize and kill cancer cells, and this technology is providing life-saving options for patients with previously incurable hematologic malignancies and beyond (16). More than ten CAR T cell products have now received regulatory approval worldwide for the treatment of B cell leukemias, lymphomas, and multiple myeloma, establishing engineered cell therapies as a standard component of modern cancer care.

In vivo AAV gene therapy is currently used for multiple rare diseases, but recently, Fractyl Health received Clinical Trial Application authorization in the Netherlands to initiate a phase I/II study of RJVA-001, an AAV-based gene therapy for type 2 diabetes. This milestone marks the first AAV gene therapy to enter clinical development for type 2 diabetes, with the goal of providing a one-time treatment that enables pancreatic cells to produce glucagon-like peptide-1 (GLP-1) endogenously (17).

Our own work illustrates how discoveries in rare disease research can reshape therapeutic paradigms for other diseases. Our initial research program on cystinosis, a rare devastating multisystem disorder, demonstrated the therapeutic potential of autologous HSC gene therapy for diseases caused by nonsecreted lysosomal transmembrane proteins, with early clinical studies showing encouraging safety and efficacy outcomes (18). This work revealed that gene-corrected HSC-derived cells can deliver functional organelles to diseased tissues throughout the body. Building on these findings, we developed HSC gene therapy programs for mucopolysaccharidosis type IIIC, a lysosomal disease, and Friedreich’s ataxia, a recessive genetic disorder that systemically affects mitochondria, and these efforts are advancing toward clinical translation. Subsequently, insights into the ability of HSCs to generate microglia-like cells that deliver genetic material to neurons in the central nervous system in Friedreich’s ataxia (19) led us to extend this approach to Alzheimer’s disease, the leading cause of dementia worldwide, where HSC transplantation prevented neuroinflammation and cognitive decline in preclinical models (20). These studies highlight how innovations emerging from rare disease research can ultimately inform therapeutic strategies for more prevalent conditions.

Taken together, these experiences highlight a central principle in translational medicine: rare diseases often serve as critical proving grounds for transformative technologies. The insights gained from understanding disease mechanisms, vector design, cellular delivery strategies, and gene editing approaches in rare disorders create a technological and conceptual foundation that can be rapidly leveraged to address more prevalent conditions. In this way, investment in rare disease research yields benefits well beyond a small number of patients; it drives innovation that ultimately expands therapeutic possibilities across the entire spectrum of human disease.

## Conflict of interest

SC is confounder, shareholder, and a member of both the scientific board and board of directors of Papillon Therapeutics Inc. SC serves as a member of the scientific review board and board of trustees of the Cystinosis Research Foundation. She is coinventor of the issued patent entitled “Methods of Treating Lysosomal Disorders” (#US-2024-0009247-A1) and inventor of the issued patent entitled “Methods for treating Mitochondrial Disorders” (#114198-3029).

## Funding support

This work is the result of NIH funding, in whole or in part, and is subject to the NIH Public Access Policy. Through acceptance of this federal funding, the NIH has been given a right to make the work publicly available in PubMed Central.

- California Institute of Regenerative Medicine grants (CLIN2-11478, TRAN1-13983 and PDEV-19140) to SC.
- NIH grants (R01NS135162 and R01AG086443) to SC.
- Cystinosis Research Foundation to SC.
- Friedreich’s Ataxia Research Alliance to SC.
- National MPS Society to SC.
- The UCSD Gene Therapy Initiative to SC.

## Figures and Tables

**Figure 1:**
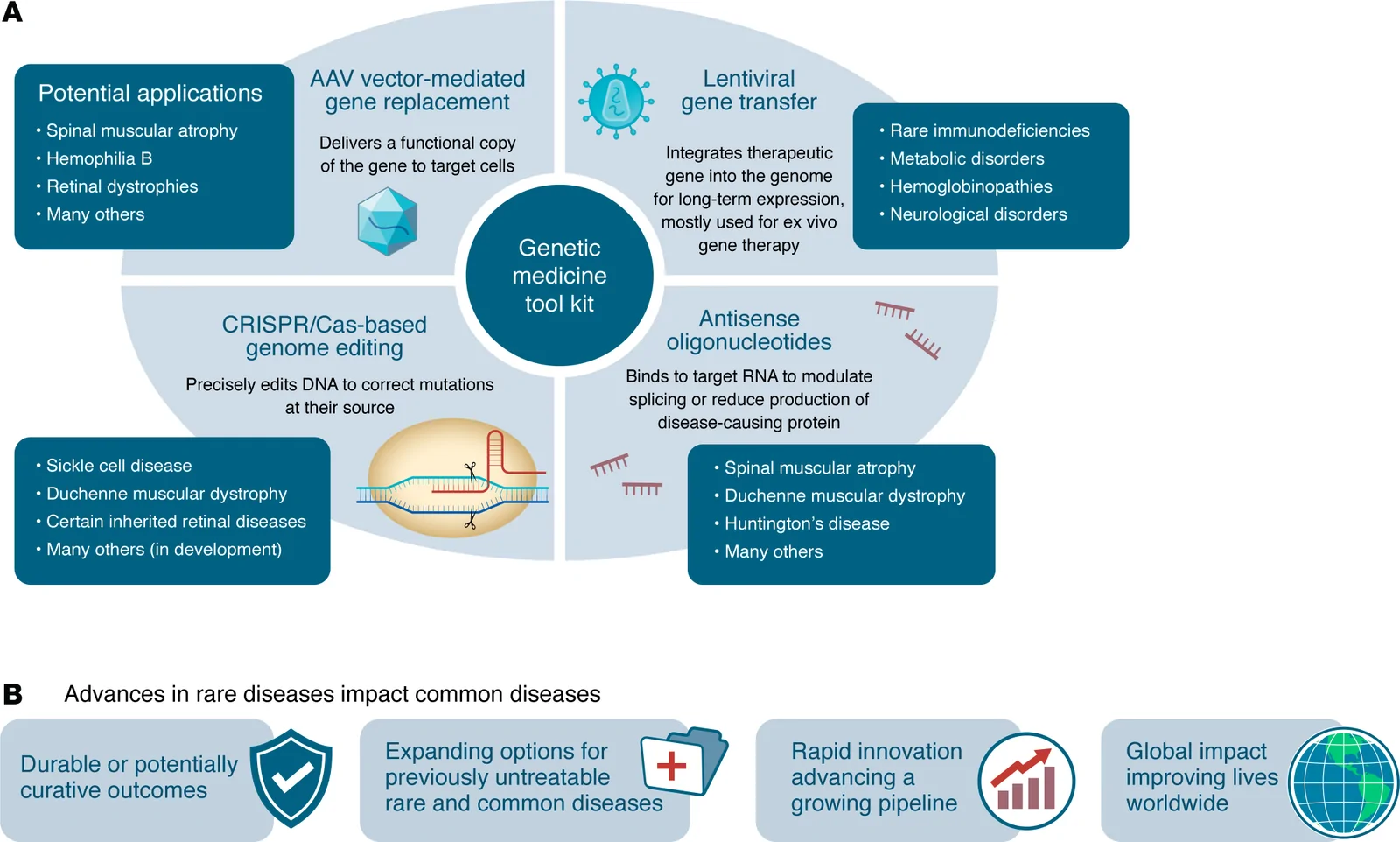
Advancing genetic medicine for treatment of rare diseases. (**A**) A brief overview of gene therapy technologies and their potential applications in rare diseases. (**B**) Rare diseases are often on the front lines of innovation in precision medicine and gene therapy, establishing a foundation for broader use of these technologies.

## Version 1 10/01/2026

Electronic publication
